# Exploring Tomato Fruit Viromes through Transcriptome Data Analysis

**DOI:** 10.3390/v15112139

**Published:** 2023-10-24

**Authors:** Yeonhwa Jo, Hoseong Choi, Bong Choon Lee, Jin-Sung Hong, Sang-Min Kim, Won Kyong Cho

**Affiliations:** 1 College of Biotechnology and Bioengineering, Sungkyunkwan University, Suwon 16419, Republic of Korea; yeonhwajo@gmail.com; 2 Plant Health Center, Seoul National University, Seoul 08826, Republic of Korea; bioplanths@gmail.com; 3 Crop Protection Division, National Academy of Agricultural Science, Rural Development Administration, Wanju 55365, Republic of Korea; leebc21@korea.kr; 4 Department of Applied Biology, Kangwon National University, Chuncheon 24341, Republic of Korea; jinsunghong@kangwon.ac.kr; 5 Crop Foundation Division, National Institute of Crop Science, Rural Development Administration, Wanju 55365, Republic of Korea

**Keywords:** tomato fruit virome, viral diversity, high-throughput sequencing, coinfections, phylogenetic analysis

## Abstract

This study delves into the complex landscape of viral infections in tomatoes (*Solanum lycopersicum*) using available transcriptome data. We conducted a virome analysis, revealing 219 viral contigs linked to four distinct viruses: tomato chlorosis virus (ToCV), southern tomato virus (STV), tomato yellow leaf curl virus (TYLCV), and cucumber mosaic virus (CMV). Among these, ToCV predominated in contig count, followed by STV, TYLCV, and CMV. A notable finding was the prevalence of coinfections, emphasizing the concurrent presence of multiple viruses in tomato plants. Despite generally low viral levels in fruit transcriptomes, STV emerged as the primary virus based on viral read count. We delved deeper into viral abundance and the contributions of RNA segments to replication. While initially focused on studying the impact of sound treatment on tomato fruit transcriptomes, the unexpected viral presence underscores the importance of considering viruses in plant research. Geographical variations in virome communities hint at potential forensic applications. Phylogenetic analysis provided insights into viral origins and genetic diversity, enhancing our understanding of the Korean tomato virome. In conclusion, this study advances our knowledge of the tomato virome, stressing the need for robust pest control in greenhouse-grown tomatoes and offering insights into virus management and crop protection.

## 1. Introduction

The tomato (*Solanum lycopersicum* L.) holds a prominent position globally due to its economic importance and versatile culinary and dietary applications [1]. However, the tomato crop faces consistent challenges from both abiotic and biotic stresses, including viral infections [2,3]. Various viruses can infect tomato plants, and the prevalence of these viruses often depends on geographical regions. Common tomato-infecting viruses include tomato mosaic virus (ToMV), tomato yellow leaf curl virus (TYLCV), tomato spotted wilt orthotospovirus (TSWV), cucumber mosaic virus (CMV), potato virus Y (PVY), tobacco mosaic virus (TMV), and tomato chlorosis virus (ToCV), all of which are known to have detrimental effects on crop yield and quality. TYLCV, a circular DNA genome, is particularly widespread and causes severe symptoms such as leaf curling, stunting, and chlorosis in tomato plants [4]. Moreover, due to high rates of mutation and genetic recombination, several TYLCV strains have been reported in the world [5]. TSWV, which has a wide host range, also infects tomatoes and causes significant yield loss around the world [6]. To overcome and prevent viral diseases, several lines resistant to TYLCV or TSWV have been developed [7,8].

The emergence of high-throughput sequencing technologies has sparked a revolution in the study of plant–virus interactions, facilitating comprehensive investigations of viral communities within plant tissues [9]. Notably, recent years have witnessed multiple studies on tomato viromes utilizing high-throughput sequencing (HTS) [10]. For instance, through small RNA sequencing, researchers identified 22 distinct viruses among 170 tomato samples in China [11]. Furthermore, large-scale HTS-based viromics endeavors in Slovenia revealed the presence of 125 viruses, including 79 novel species, in tomatoes and weeds [12]. Additionally, comparative virome analyses were conducted between cultivated *Solanum lycopersicum* and wild *Solanum nigrum* [13], and HTS was employed on a pool of 106 tomato and 53 pepper field-grown plants in Tennessee, USA [14].

In Korea, the cultivation of tomato plants has steadily expanded in tandem with economic growth. Notably, a diverse array of viruses infecting tomatoes in Korea have been previously documented. Among the identified viruses affecting tomatoes in Korea are TYLCV, CMV, ToCV, ToMV, TSWV, southern tomato virus (STV), pepper mottle virus (PepMoV), and tomato bushy stunt virus (TBSV) [15,16,17,18,19,20].

While numerous researchers have delved into tomato viromes, none have specifically examined viromes within tomato fruits in their studies. Additionally, there have been no comprehensive studies conducted on tomato viromes in Korea as per our knowledge. RNA sequencing data prove invaluable in dissecting plant and fungal viromes, uncovering a plethora of novel insights [21,22,23,24]. Before embarking on our investigation of the tomato virome in Korea, we screened tomato transcriptomes derived from Korea in the public database to gather foundational information on tomato viruses in the region. Among these datasets, one study, focused on tomato fruits subjected to sound treatment, yielded valuable insights into the tomato virome, as confirmed by our preliminary analysis [25].

The primary objective of this study is to analyze tomato viromes using existing transcriptome data, with a specific emphasis on fruit viromes in tomatoes. This investigation encompasses assessing viral abundance, diversity, and community dissimilarities, visualizing sample relationships through principal coordinates analysis (PCoA), annotating viral genomes, and conducting phylogenetic analysis. Furthermore, we also explore the potential influence of sound treatment on viral dynamics in tomatoes. Ultimately, this study aims to enrich our comprehension of viral diversity, abundance, and interactions within tomato fruit transcriptomes.

## 2. Materials and Methods

### 2.1. Sample Collection and Library Preparation

For this study, we employed previously published tomato transcriptome data to investigate the virome. A comprehensive description of the sample collection and treatment can be found in the previous study [25]. In summary, mature green tomatoes (*Solanum lycopersicum* L. cultivar Dotaerang) were obtained from commercial glasshouses in two distinct regions, Jeongeup and Yong-In, Korea. These tomatoes were visually assessed to ensure uniformity. The harvested fruit samples underwent a 6-h exposure to a 1 kHz sound wave at 80 dB. Subsequently, samples were collected at four distinct time points: 6 h (6H), 2 days (2D), 5 days (5D), and 7 days (7D) post-treatment. Three biological replicates were obtained for each condition and time point, resulting in a total of 24 samples. Total RNA extraction from the fruit samples was performed using the RNeasy Mini Kit (Qiagen, Valencia, CA, USA) following the manufacturer’s instructions. The mRNA libraries were prepared and subjected to paired-end sequencing (2 × 150 bp) using the HiSeq-2000 platform (Illumina, San Diego, CA, USA), resulting in 24 transcriptomes covering control samples and sound-treated samples.

### 2.2. Data Processing and Transcriptome Assembly

Raw sequencing data associated with accession number PRJNA416331 (SRR6234973—SRR6234988 and SRR7668108—SRR7668115) were retrieved from the National Center for Biotechnology Information (NCBI) Sequence Read Archive (SRA) database. The SRA data were converted into FASTQ format using the SRA-Toolkit (https://hpc.nih.gov/apps/sratoolkit.html) (accessed on 11 November 2021). Subsequently, the raw FASTQ files underwent quality control, including read trimming and the removal of low-quality reads, using the BBDuk program (https://jgi.doe.gov/data-and-tools/software-tools/bbtools/bb-tools-user-guide/bbduk-guide/) (accessed on 11 November 2021). The resulting high-quality reads were utilized for de novo transcriptome assembly, performed with Trinity version v2.13.2 using default parameters [26] (accessed on 11 November 2021).

### 2.3. Virus Identification

Transcriptome contigs from each library were subjected to a BLASTX search against the viral protein database obtained from NCBI (https://www.ncbi.nlm.nih.gov/genome/viruses/) with an E-value cutoff of 1E-10 (accessed on 11 November 2021). Contigs showing sequence similarity to viral proteins were further analyzed through a BLASTX search against the non-redundant (NR) protein database at NCBI to precisely identify viral contigs. The identified viral contigs were then categorized by their corresponding virus species. For alignment of raw sequence reads with reference viral genomes, we used the BWA aligner version 0.7.17 with default parameters [27] (accessed on 11 November 2021). To quantify viral abundance in individual samples, we calculated coverage, viral reads, and fragments per kilobase of transcript per million (FPKM) using the eXpress program (https://pachterlab.github.io/eXpress/manual.html) (accessed on 11 November 2021) based on the SAM file.

### 2.4. Analysis of Viral Proportion and Abundance

To assess the relative abundance of identified viruses in each tomato sample, we calculated the proportion of viral reads relative to the total reads, providing insights into viral replication within individual samples. Additionally, to account for variations in viral genome sizes and sequencing depth, we normalized viral abundance using the Fragments Per Kilobase of transcript per Million mapped reads (FPKM) method. FPKM values were computed for each identified virus in every sample, offering a more accurate assessment of the proportion of individual viruses within the transcriptomes. These FPKM values were then used to analyze the relative viral proportion in each sample.

### 2.5. Investigation of Sound Treatment Effects on Tomato Viromes

Our primary objective was to investigate the impact of sound treatment on viral dynamics within tomato fruits. To achieve this, we used eight experimental conditions, comprising four control samples and four sound-treated samples collected at various time points. The proportion of viral replications in each condition was determined by pooling data from the three biological replicates for each condition.

### 2.6. Alpha and Beta Diversity Analysis

Alpha diversity analysis was conducted to assess the diversity of identified viruses within and among different conditions, biological replicates, and time points. We employed the Shannon and Simpson diversity indices for this analysis. Beta diversity analysis aimed to evaluate dissimilarities in viral communities across different conditions, time points, and replicates, and we used a Permutational Multivariate Analysis of Variance (PERMANOVA) to determine the significance of these dissimilarities. Both alpha and beta diversity analyses were performed using the Mian platform (https://miandata.org/) [28] (accessed on 1 August 2023).

### 2.7. Principal Coordinate Analysis (PCoA)

PCoA was conducted to visualize the relationships between individual samples based on their viral compositions using the Mian platform (https://miandata.org/) (accessed on 2 September 2023) [28]. This analysis provided insights into the clustering of samples according to experimental conditions, time points, and replicates.

### 2.8. Viral Genome Annotation

From the obtained viral contigs, we selected those covering complete open reading frames (ORFs), resulting in complete or nearly complete sequences for 4 CMV RNA segments, 2 ToCV RNA2 segments, and 14 STV genomes. The ORFfinder program, available at the NCBI website (https://www.ncbi.nlm.nih.gov/orffinder/) (accessed on 11 November 2021), was utilized to predict ORFs within the viral contigs. To ensure consistency, reverse sequences were converted using the DNA Reverse Complement Calculator (https://jamiemcgowan.ie/bioinf/complement.html) (accessed on 11 November 2021). Subsequently, we conducted a BLASTX search against a non-redundant protein database to identify various viral genomic attributes, including conserved motifs and potential functional elements, by comparing them with previously characterized viral genomes. All complete viral genome sequences obtained were deposited in NCBI’s GenBank with the following accession numbers: 14 STV genomes (OR596862–OR596875), 4 CMV segments (OR596876–OR596879), and 2 ToCV RNA2 segments (OR596880–OR596881).

### 2.9. Phylogenetic Analysis

For the construction of phylogenetic trees, we obtained all available viral genome sequences from the NCBI GenBank database. Viral sequences were aligned using MAFFT version 7 with the auto option [29]. Subsequently, sequence trimming was performed to remove the 5′ and 3′ untranslated regions (UTRs) using trimAl version 1.2 [30]. Model selection for the aligned sequences was conducted, followed by the construction of phylogenetic trees using IQ-TREE version 1.6.12, utilizing the maximum likelihood method with 1000 bootstrap replicates [31]. The resulting phylogenetic trees were visualized and edited using Figtree version 1.4.4 (http://tree.bio.ed.ac.uk/software/figtree/) (accessed on 2 September 2023).

## 3. Results

### 3.1. Viral Contig Identification and Genomic Classification

In this study, we used previous RNA sequencing experiments which investigated transcriptional changes in tomato fruits in response to sound wave treatment [25]. The research encompasses 24 distinct libraries, each assigned a unique accession number, and includes two distinct sample conditions, four time points, and three replicates (Table 1). Control samples, denoted by “C,” were collected at various time points, specifically six hours (6H), two days (2D), five days (5D), and seven days (7D), and were further categorized into replicates R1, R2, and R3. Similarly, samples subjected to sound wave treatment, referred to as “sound” samples, were also collected at these time points with their respective replicates. For instance, consider library T13, which corresponds to a control sample collected at 6 h, replicate R1, with its RNA sequencing data identified by the accession number SRR6234985. This extensive dataset provides valuable insights into the dynamics of the tomato fruit virome under different experimental conditions and over various time intervals.

**Figure 1:**
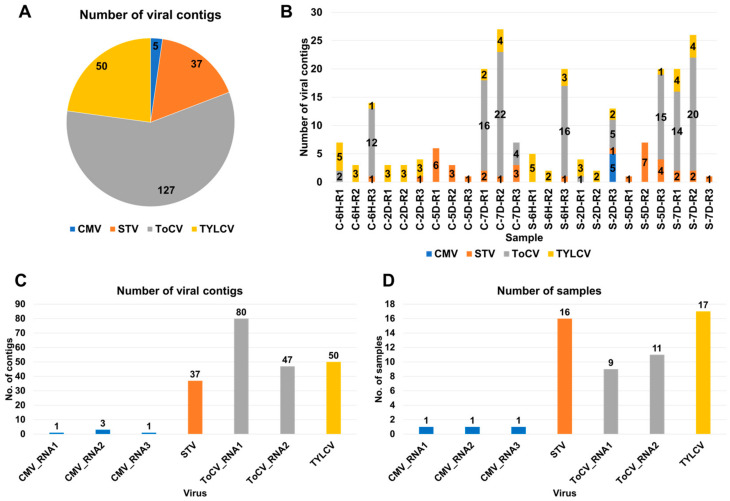
Overview of identified viruses based on viral contigs. (**A**) Distribution of viral contigs among identified viruses across 24 tomato transcriptomes; each virus is represented by a unique color. (**B**) Number of viral contigs detected in individual samples; the identified viruses are color-coded. (**C**) Distribution of viral contigs assigned to each identified virus. (**D**) Occurrence of identified viruses in the samples; for CMV and ToCV, the number of viral contigs assigned to each RNA segment is also displayed.

Raw data were downloaded from the SRA database at NCBI (Table S1) and subjected to data processing followed by de novo transcriptome assembly, as described in materials and methods. After the BLASTX search against NR database, we identified a total of 219 viral contigs assigned to 4 different viruses in 24 tomato transcriptomes (Table S2 and Figure 1A). Among these, ToCV had the highest number of associated contigs (127 contigs), followed by STV with 50 contigs, TYLCV with 30 contigs, and CMV with 5 contigs. The number of identified viral contigs within each sample ranged from one to twenty-seven contigs (Figure 1B).

To further classify the viral contigs according to virus genome segments, we examined the distribution (Figure 1C). CMV consists of three RNA segments, namely RNA1 (one contig), RNA2 (three contigs), and RNA3 (one contig). ToCV, on the other hand, is composed of two RNA segments, RNA1 (80 contigs) and RNA2 (47 contigs). CMV was only identified in a single sample (S-2D-R3) (Figure 1D). However, TYLCV was identified in 17 samples, while STV was identified in 16 samples. ToCV RNA1 was detected in nine samples, and ToCV RNA2 was detected in eleven samples.

### 3.2. Viral-Read Proportions and Co-Infection Complexity

**Figure 2:**
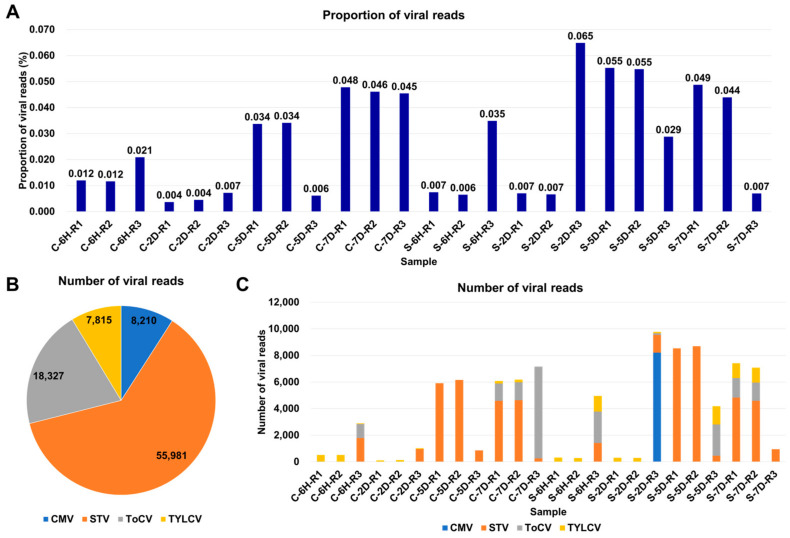
Overview of identified viruses based on viral reads. (**A**) Proportion of viral reads in each sample. (**B**) Distribution of viral reads among identified viruses across 24 tomato transcriptomes, color-coded according to the respective viruses. (**C**) Number of viral reads assigned to each identified virus in individual samples.

The proportion of viral reads within a transcriptome can provide valuable insights into viral replication within infected samples. To investigate this, we examined the viral-read proportions in each sample (Table S2). In the tomato samples, these proportions ranged from 0.004% to 0.065%, indicating a very low level of viral presence in the tomato samples (Figure 2A). Southern tomato virus (STV) was the most predominant virus based on the number of viral reads, with 55,981 reads, followed by tomato chlorosis virus (ToCV) with 18,327 reads, tomato yellow leaf curl virus (TYLCV) with 7815 reads, and cucumber mosaic virus (CMV) with 8210 reads (Figure 2B). The number of viral reads within each sample exhibited variation, ranging from 110 to 9766 reads (Figure 2C). Notably, in 10 samples, the number of viral reads was less than 1000 reads. Furthermore, we observed that half of the samples were infected by multiple viruses. Specifically, five samples were coinfected by two viruses, while six samples were coinfected by three viruses. Sample S-2D-R3 exhibited the highest complexity, being coinfected by four viruses.

### 3.3. Viral Abundance and Genome Segment Analysis

**Figure 3:**
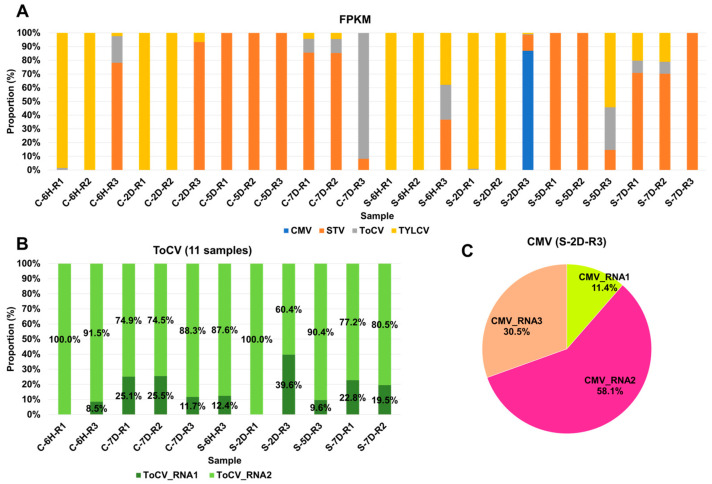
Proportion of identified viruses in each sample based on FPKM values. (**A**) Proportion of identified viruses in each sample based on FPKM values; different colors represent the identified viruses. (**B**) Proportion of ToCV RNA1 and RNA2 segments in the infected samples. (**C**) Pie chart displaying the proportion of three CMV RNA segments.

In general, viruses with larger genomes tend to generate a higher number of viral reads. Therefore, it is crucial to estimate the viral abundance through normalization. To achieve this, we computed FPKM values for the identified viruses in each sample (Table S2). Based on these FPKM values, we analyzed the proportion of individual viruses in each sample (Figure 3A). TYLCV and STV emerged as the most dominant viruses across the samples. TYLCV was the dominant virus in at least nine samples, while STV held dominance in twelve samples. In specific cases, such as C-7D-R3 and S-2D-R3, ToCV and CMV were the dominant viruses, respectively. In the case of S-2D-R3, which was coinfected by four viruses, CMV was the dominant presence. Several samples, including C-6H-R3, S-6H-R3, S-5D-R3, S-7D-R1, and S-7D-R2, were coinfected by three viruses—STV, ToCV, and TYLCV. The proportions of each virus varied depending on the specific plant sample. ToCV and CMV genomes consist of multiple RNA segments, making it interesting to explore whether similar viral replication can be attributed to individual RNA segments. Concerning ToCV, RNA1 was predominantly present, ranging from 60.4% to 100%, in contrast to RNA2, which ranged from 0% to 39.6% (see Figure 3B). In the case of CMV, RNA2 was the dominant segment, accounting for 58.1%, followed by RNA3 at 30.5%, and RNA1 at 11.4%.

### 3.4. Impact of Sound Treatment on Viral Dynamics and Replication

**Figure 4:**
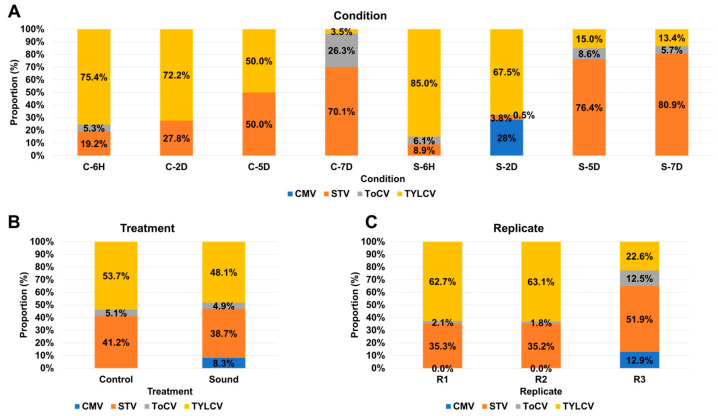
Viral abundance comparison based on FPKM values. (**A**) Comparative viral abundance in eight distinct conditions. The FPKM values from replicates were aggregated for each condition. (**B**) Viral abundance comparison between control and sound-treated samples. We examine the differences in viral abundance between control samples and those subjected to sound treatment. (**C**) Viral abundance comparison across replicates. We analyze the variation in viral abundance among different replicates to assess result consistency.

This study was initially designed to investigate transcriptional changes in tomato fruits in response to sound treatment. To achieve this, we utilized eight different experimental conditions, consisting of four control samples without sound treatment and four samples subjected to sound treatment at various time points: six hours (6H), two days (2D), five days (5D), and seven days (7D). The proportion of viral replications in each condition was assessed by combining three replicates for each condition (Figure 4A). In both control and sound-treated samples, TYLCV dominated at both 6H and 2D time points. However, over time, the proportion of TYLCV decreased, while the proportion of STV increased significantly. The presence of ToCV was notable only in the C-7D condition, remaining below 10% in all other conditions. CMV was exclusively detected in the S-2D condition, comprising 28% of the viral population. Subsequently, we compared the proportion of viral replications between the control and sound-treated samples (Figure 4B). In both conditions, TYLCV remained the dominant virus, followed by STV. Notably, the sound-treated samples exhibited the presence of four different viruses, while the control samples had only three viruses. There were no significant differences in viral proportions between the two conditions, except for CMV. Further investigation of viral proportions among different replicates was conducted (Figure 4C). Interestingly, R1 and R2 exhibited similar viral compositions, with TYLCV being the dominant virus, followed by STV and ToCV. Conversely, in the case of R3, among the four identified viruses, STV was the most dominant, followed by TYLCV, CMV, and ToCV.

### 3.5. Alpha and Beta Diversity Analysis of Viral Communities

**Figure 5:**
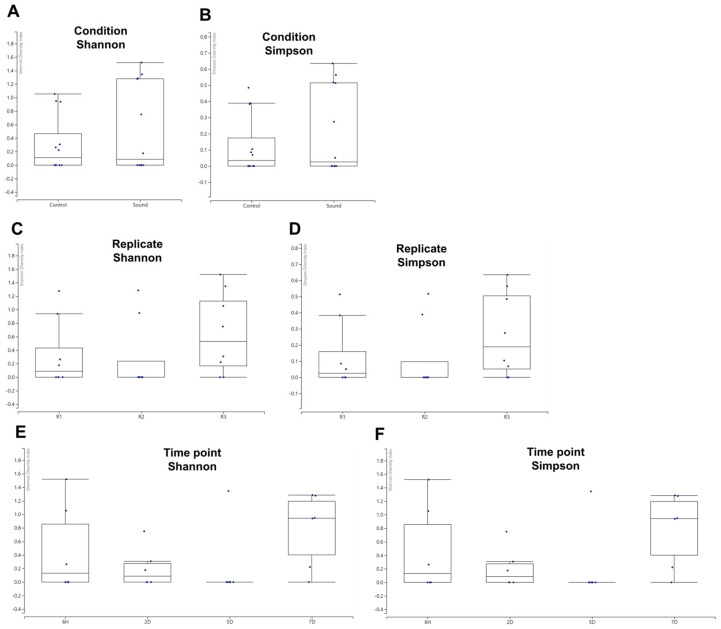
Alpha diversity of identified viruses in various conditions. (**A**,**B**) Alpha diversity comparison between control and sound-treated samples. The graph presents the alpha diversity of identified viruses in control samples versus those exposed to sound treatment, using both Shannon (**A**) and Simpson (**B**) diversity indices. (**C**,**D**) Alpha diversity among replicates. The alpha diversity of identified viruses was evaluated among different replicates, employing both Shannon (**C**) and Simpson (**D**) diversity indices. (**E**,**F**) Alpha diversity at four different time points. The alpha diversity of identified viruses was examined across four distinct time points, utilizing both Shannon (**E**) and Simpson (**F**) diversity indices.

While we identified a limited number of viruses, we proceeded to analyze the alpha diversity of these identified viruses across various conditions using the viral reads we had identified. The box plots revealed that the sound-treated condition exhibited a high degree of variance among samples. However, no significant differences in alpha diversity were observed between the two conditions when assessed using Shannon and Simpson indices (Figure 5A,B). When examining the alpha diversity among different biological replicates, R1 and R2 displayed similar box plot shapes, while R3 exhibited a high degree of variance among samples (Figure 5C,D). Nevertheless, no significant differences in alpha diversity were observed among the replicates. Among the four different time points, samples collected at 5D exhibited relatively similar viromic patterns, whereas those from 6H and 7D displayed a high degree of variance (Figure 5E,F). Once again, there were no significant differences in alpha diversity observed among the four time points.

**Figure 6:**
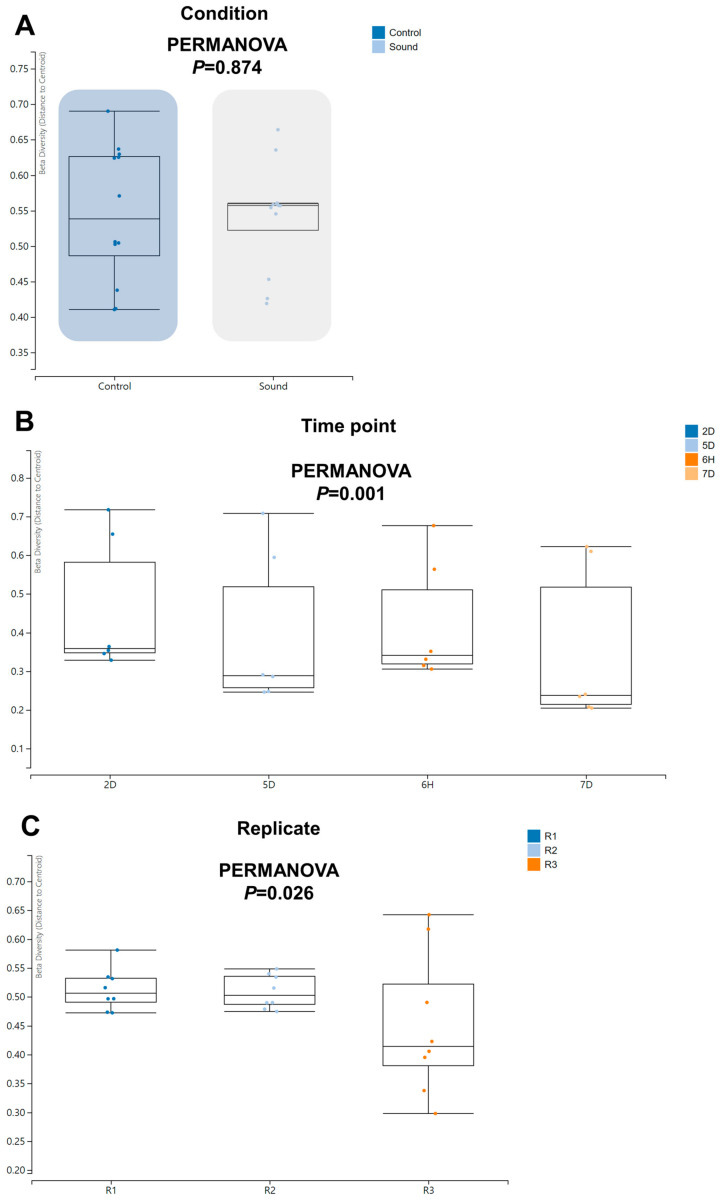
Beta diversity of identified viruses across varied conditions. Beta diversity analysis was performed using PERMANOVA to assess the dissimilarity in viral communities under different conditions (**A**), across four time points (**B**), and among three replicates (**C**).

Moving forward, we proceeded to investigate beta diversity to determine if there were any significant differences among the various conditions. Notably, we did not observe a significant difference in beta diversity between the control and sound-treated conditions (Figure 6A). However, we did identify a significant difference in beta diversity among the four time points (*p* = 0.001) and among the replicates (*p* = 0.026).

**Figure 7:**
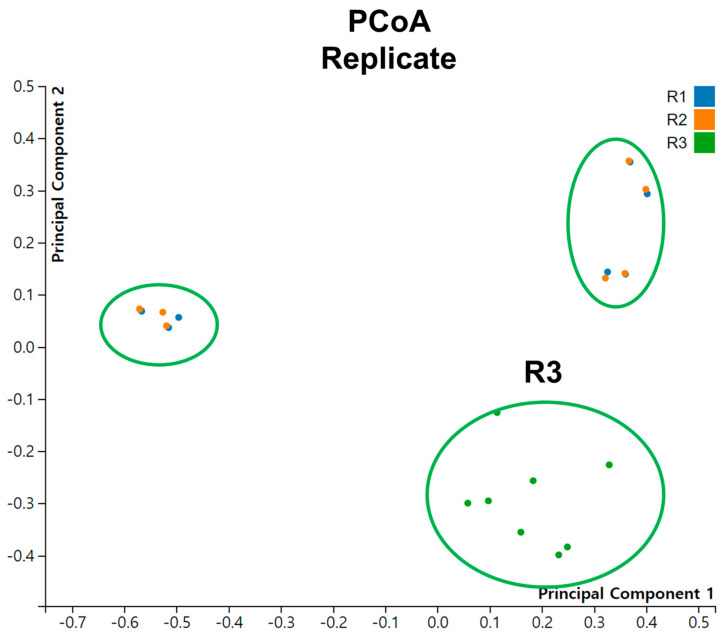
Principal coordinate analysis (PCoA) of viromes for individual samples across replicates.

We conducted Principal Coordinate Analysis (PCoA) on the dataset comprising 24 tomato viromes (Figure 7). Interestingly, we did not observe distinct groupings of samples based on different conditions or time points. However, when considering the replicates, it is notable that samples from R3 formed a distinct grouping.

### 3.6. Phylogenetic Analysis Using Obtained Viral Genomes

**Figure 8:**
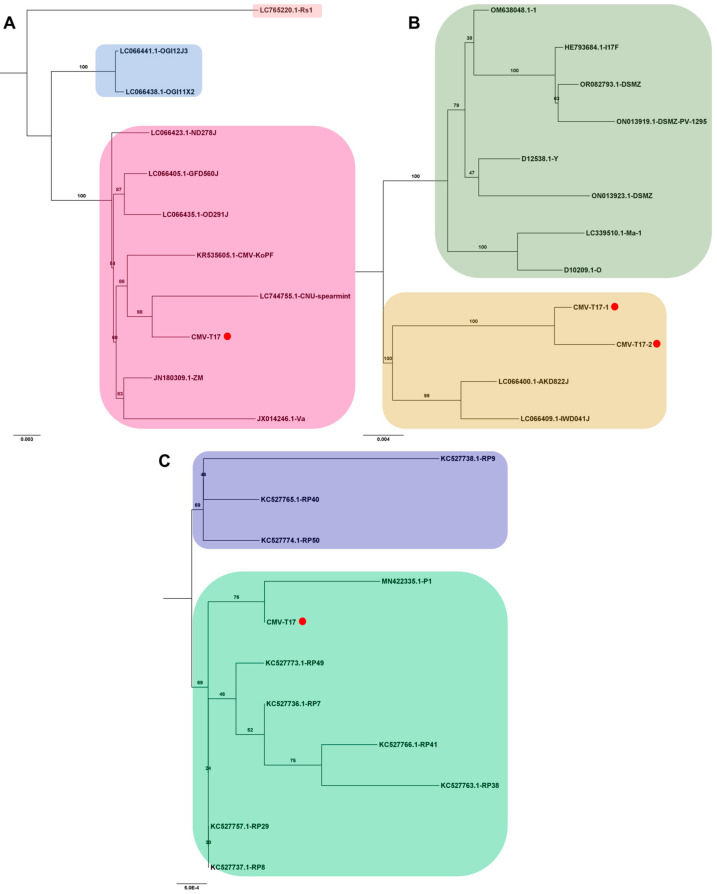
Phylogenetic analysis of assembled CMV RNA segments and known CMV isolates. The phylogenetic relationship of the assembled CMV RNA segments with known CMV isolates was analyzed. Phylogenetic trees for CMV RNA1 (**A**), RNA2 (**B**), and RNA3 (**C**) were constructed using the maximum likelihood method, with 1000 bootstrap replicates to assess the robustness of the tree topologies. Red dots on the trees represent the assembled CMV RNA fragments obtained in this study. To ensure the accuracy of our analysis, we included only ten known CMV genome segments that exhibited high sequence similarity to the assembled CMV genome segments from this study in the phylogenetic tree construction.

Several viral genomes were obtained from tomato transcriptomes in this study (Table S3). The viral genomes covering entire open reading frames were employed to analyze their phylogenetic relationships with known viral isolates. For CMV, one RNA1 genome, two RNA2 genomes, and one RNA3 genome were obtained exclusively from the T17 sample. Due to the abundance of available CMV genomes, only the top ten viral genomes showing the highest sequence similarity via BLASTN search were selected for analysis. In the case of CMV RNA1, isolate T17 exhibited a close relationship with the known CNU-spearmint isolate (GenBank LC744755.1) from Mentha spicata in Korea (Figure 8A). For CMV RNA2, two complete genomes closely resembled known isolates AKD822J (LC066400.1) and IWD041J (LC066409.1) from Raphanus sativus in Japan (Figure 8B). The CMV RNA3 isolate T17 clustered with the P1 strain (GenBank MN422335.1) from pepper (*Capsicum annuum*) in the Republic of Korea (Figure 8C).

**Figure 9:**
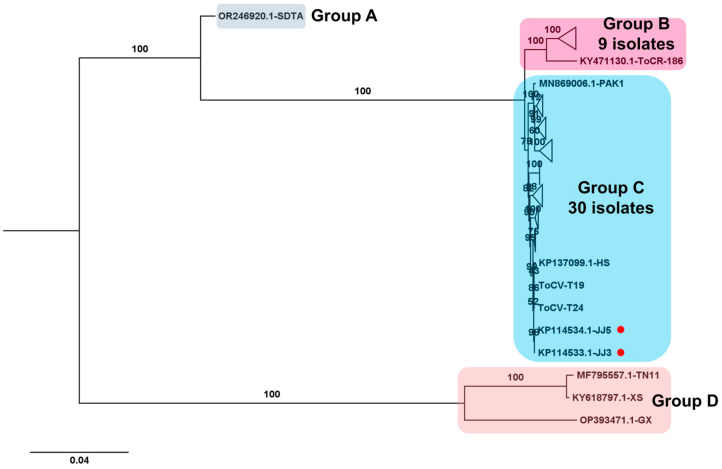
Phylogenetic analysis of assembled ToCV RNA2 segments and known ToCV Isolates. The phylogenetic relationship between two assembled ToCV RNA2 segments in this study and known ToCV isolates was assessed. The phylogenetic tree of ToCV was constructed using the maximum likelihood method with 1000 bootstrap replicates. The assembled ToCV RNA2 fragments in this study are denoted by red dots. For the construction of the phylogenetic tree, ToCV RNA2 segment sequences covering entire ORFs, sourced from GenBank, were utilized. To simplify the phylogenetic tree, certain clades were compressed and represented as triangles. The complete phylogenetic tree can be found in Figure S1.

Regarding the ToCV genome, composed of two RNA segments, two complete ToCV RNA2 segments were obtained from samples T19 and T24. The phylogenetic tree comprising 43 complete ToCV RNA2 sequences revealed four major groups (Figure 9). Group A contained the isolate SDAT (GenBank OR246920.1) from tomato in China, genetically distinct from other isolates. Group B included nine isolates, while group C comprised thirty isolates, with isolates T19 and T24 from this study falling into group C. Additionally, three isolates, including two (TN11 and XS) from Taiwan and one (GX) from China, were categorized into group D.

**Figure 10:**
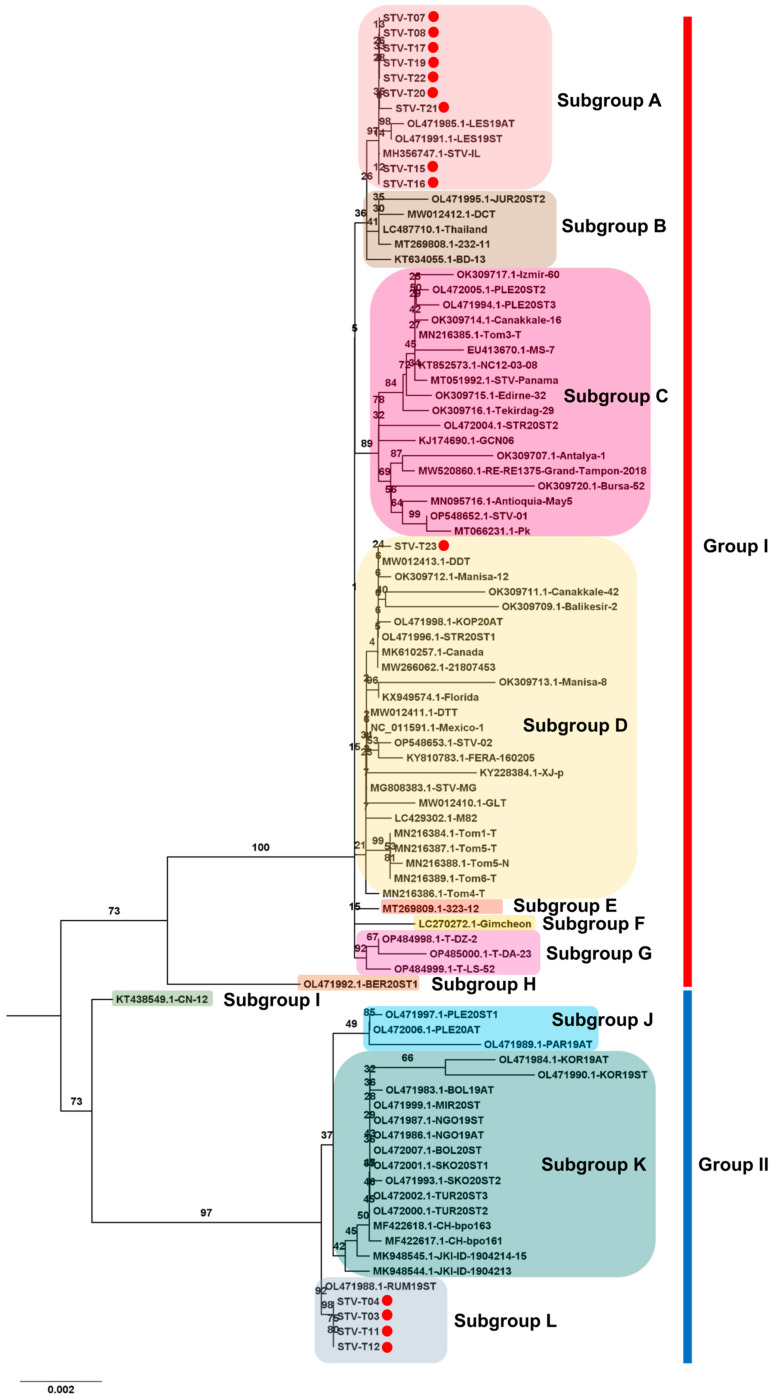
Phylogenetic analysis of assembled STV genomes and known STV Isolates. The phylogenetic relationship between 14 assembled STV genomes in this study and known STV isolates was examined. The phylogenetic tree of STV was generated using the maximum likelihood method with 1000 bootstrap replicates. The assembled STV genomes in this study are represented by red dots. For the construction of the phylogenetic tree, STV genome sequences covering entire ORFs, sourced from GenBank, were employed.

For STV, 14 complete genomes were obtained. The phylogenetic tree, using a total of 89 complete genome sequences for STV, identified two main STV groups, Group I and Group II (Figure 10). Intriguingly, ten STV isolates from this study belonged to Group I, while four STV isolates belonged to Group II. Group I further divided into eight subgroups, A to H. Nine isolates from this study (T07, T08, T15, T16, T17, T19, T20, T21, and T22) clustered together with three known isolates (LES19AT and LES19ST from Serbia, and STV-IL from Israel). STV isolate T23, closely related to DCT from Vietnam, fell into subgroup D, the largest subgroup. In Group II, four subgroups, I to L, were identified. The four isolates from this study (T03, T04, T11, and T12) grouped with RUM19ST from Serbia in subgroup L. Additionally, several subgroups, such as E, F, H, and I, were represented by a single STV isolate.

## 4. Discussion

To date, the detection of viruses infecting tomato plants in Korea has primarily relied on polymerase chain reaction (PCR) or reverse transcriptase PCR (RT-PCR) using virus-specific primers [15,20]. However, PCR-based methods can only identify known viruses and do not provide complete viral genome sequences. In contrast, high-throughput sequencing (HTS)-based approaches offer a broader range of information for characterizing infecting viruses.

In our study of 24 tomato fruit transcriptomes, we employed de novo transcriptome assembly to accurately identify viral species. This approach successfully led to the identification of four viruses: ToCV, STV, TYLCV, and CMV. Previously, ToCV and TYLCV infections in tomatoes in Korea, the United States, and Spain had been identified using PCR-based methods by different research groups [16,32,33,34]. Additionally, the first report of STV infecting tomatoes in Korea was in 2015 [17]. The previous study revealed that all STV-infected tomatoes were coinfected with TYLCV [17], suggesting that the most common viruses infecting commercially grown tomatoes in Korea might be STV, ToCV, and TYLCV.

Prior research on tomato-infecting viruses in Korea had predominantly focused on individual viruses rather than on the broader tomato virome. In comparison to these earlier studies, our research represents the first comprehensive investigation of the tomato virome in Korea, uncovering multiple viruses affecting tomatoes grown in the region.

We conducted various analyses to explore the virome, taking into account several parameters associated with viruses. One key observation was the correlation between the number of viral contigs obtained and viral abundance, as well as genome size. Notably, ToCV yielded the highest number of viral contigs due to its sizable genome composed of two RNA segments. However, when considering viral reads, STV emerged as the most abundant virus. Coinfections were a common occurrence, with several samples harboring multiple viruses, with TYLCV and STV frequently co-occurring. To account for variations in viral replication, we employed the FPKM method to estimate viral abundance, revealing TYLCV and STV as the dominant viruses. Notably, significant differences in their viral abundance were observed between early and late time points, with STV gradually becoming more dominant. Furthermore, we observed variability in viral replication patterns within viruses with multiple segments, such as ToCV.

It is noteworthy that all 24 samples were infected by at least one virus, with many displaying multiple viral infections, underscoring the prevalence of coinfections in tomato plants. This aligns with previous reports suggesting that the coinfection of multiple viruses can induce severe disease symptoms, whereas a single virus infection may not result in apparent symptoms in tomatoes [35]. Typically, most commercial tomato plants in Korea are grown in greenhouses. Without effective management of insect vectors within these greenhouses, there is a high likelihood of frequent occurrences of viruses, such as TYLCV and ToCV, which are known to be transmitted by whiteflies, and CMV, which is transmitted by aphids [36,37]. Proper pest control measures are crucial to mitigate the risk of viral infections in greenhouse-grown tomatoes.

A previous study emphasized the importance of considering growth stages and plant tissues when detecting tomato brown rugose fruit virus, suggesting the collection of young leaves, sepals, and fruits for virus-detection purposes [38]. Similarly, our current study has successfully identified four distinct viruses in tomato fruits. However, it is worth noting that the levels of viral replication within tomato fruits were relatively low, indicating that fruit tissue may not be an optimal environment for extensive virus replication. Consequently, the viral symptoms observed in the samples were likely mild. Additionally, it’s possible that the tomato fruits used in this study had been stored for an extended period after harvesting to observe their maturation, which could have affected the freshness of the plant materials.

Originally, our study aimed to investigate changes in the tomato fruit transcriptome following sound wave treatment. Ideally, virus-free samples would have been preferable for such research. However, it is not uncommon for plant biologists to overlook the presence of viruses in their samples, especially when viral symptoms are mild or absent. This underscores the importance of considering potential virus infections in studies of this nature.

Regarding alpha diversity analysis, it did not yield significant differences, likely due to the limited sample size and the number of infecting viruses. Nevertheless, beta diversity analysis, using PERMANOVA, revealed significant variations in viromes across different time points and among replicates. These variations were consistent with the observed changes in viral abundance over time, particularly for TYLCV and STV.

The utilization of high-throughput sequencing provided us with the capability to obtain complete or nearly complete viral genomes. Specifically, we successfully sequenced four segments of CMV, two segments of ToCV RNA2, and a remarkable total of fourteen genomes of STV. When we conducted phylogenetic analyses, it became apparent that CMV isolate T17 may have originated in Korea, as it displayed sequence similarities with known Korean isolates found in various host species.

One of the most intriguing and noteworthy findings of our study was the division of the 14 STV genomes into two genetically distinct groups. This underscores the significant genetic diversity present among STV isolates. Additionally, it’s worth mentioning that STV is known to be transmitted at a high rate through seeds [39]. Therefore, it is conceivable that seed transmission could be a contributing factor to the spread of STV in Korea. Consequently, it becomes imperative to implement measures to prevent the dissemination of STV via seeds.

Furthermore, our analysis of the samples labeled as R1, R2, and R3 revealed significant differences. Previous studies had reported that samples were collected from two different regions, and the SRA accession numbers were not assigned sequentially between samples in R1 and R2 compared to those in R3. These findings unequivocally indicate that R3 samples were not collected from the same region as R1 and R2, suggesting the existence of distinct virome communities based on geographical regions. This result suggests the potential application of virome analysis in forensic investigations [40].

## 5. Conclusions

This study provides a comprehensive examination of the tomato virome in Korea, yielding several key findings. We identified and classified four distinct viruses, with ToCV exhibiting the highest contig count, followed by STV, TYLCV, and CMV. Coinfections were prevalent, underscoring the risk posed by multiple viruses in tomato plants. Our analyses revealed low levels of viral presence within the transcriptomes, with STV emerging as the most predominant virus. We also explored viral abundance and the contributions of RNA segments to replication. While our original intent was to study the impact of sound treatment on tomato fruit transcriptomes, the presence of viruses in the samples highlights the importance of considering viral infections in plant research. Moreover, geographical variations in virome communities were observed, hinting at potential forensic applications. Phylogenetic analysis provided insights into viral origins and genetic diversity. In summary, this study enhances our understanding of the tomato virome in Korea, emphasizing the need for effective pest control in greenhouse-grown tomatoes and informing strategies for virus management and crop protection.

## Figures and Tables

**Table 1:** Summary of RNA sequencing data for different libraries, sample conditions, and time points. Index: unique identifier for each entry in the table. Library name: the name of the RNA library. Sample name: the name of the RNA sample. Condition: the experimental condition of the sample (Control or Sound). Time point: the time point at which the sample was taken (e.g., 6H for six hours, 2D for two days, 5D for five days, 7D for seven days). Replicate: the replicate number for the sample. Acc. No.: the accession number or identifier for the RNA sequencing data.

Index	Library Name	Sample Name	Condition	Time Point	Replicate	Acc. No.
1	T13	C-6H-R1	Control	6H	R1	SRR6234985
2	T14	C-6H-R2	Control	6H	R2	SRR6234986
3	T23	C-6H-R3	Control	6H	R3	SRR7668114
4	T09	C-2D-R1	Control	2D	R1	SRR6234981
5	T10	C-2D-R2	Control	2D	R2	SRR6234982
6	T21	C-2D-R3	Control	2D	R3	SRR7668112
7	T11	C-5D-R1	Control	5D	R1	SRR6234983
8	T12	C-5D-R2	Control	5D	R2	SRR6234984
9	T22	C-5D-R3	Control	5D	R3	SRR7668113
10	T15	C-7D-R1	Control	7D	R1	SRR6234987
11	T16	C-7D-R2	Control	7D	R2	SRR6234988
12	T24	C-7D-R3	Control	7D	R3	SRR7668115
13	T05	S-6H-R1	Sound	6H	R1	SRR6234977
14	T06	S-6H-R2	Sound	6H	R2	SRR6234978
15	T19	S-6H-R3	Sound	6H	R3	SRR7668110
16	T01	S-2D-R1	Sound	2D	R1	SRR6234973
17	T02	S-2D-R2	Sound	2D	R2	SRR6234974
18	T17	S-2D-R3	Sound	2D	R3	SRR7668108
19	T03	S-5D-R1	Sound	5D	R1	SRR6234975
20	T04	S-5D-R2	Sound	5D	R2	SRR6234976
21	T18	S-5D-R3	Sound	5D	R3	SRR7668109
22	T07	S-7D-R1	Sound	7D	R1	SRR6234979
23	T08	S-7D-R2	Sound	7D	R2	SRR6234980
24	T20	S-7D-R3	Sound	7D	R3	SRR7668111

## Data Availability

The raw datasets (SRR6234973—SRR6234988 and SRR7668108—SRR7668115) associated with the accession number PRJNA416331 are available from the Sequence Read Archive (SRA) database at the National Center for Biotechnology Information (NCBI).

## Supplementary Materials

The following supporting information can be downloaded at: https://www.mdpi.com/article/10.3390/v15112139/s1, Table S1: Summary of RNA sequencing data for tomato fruit virome study; Table S2: Summary of virus identification from 24 tomato transcriptomes; Table S3: Summary of assembled viral genomes from 24 tomato fruit transcriptomes; Figure S1: The full phylogenetic tree of assembled ToCV RNA2 segments and established ToCV isolates.
