# Supplementary figures and images for: Exploring Tomato Fruit Viromes through Transcriptome Data Analysis
Source: Viruses. 2023 Oct 24;15(11):2139. doi: 10.3390/v15112139 (PMC10674750; doi:10.3390/v15112139)

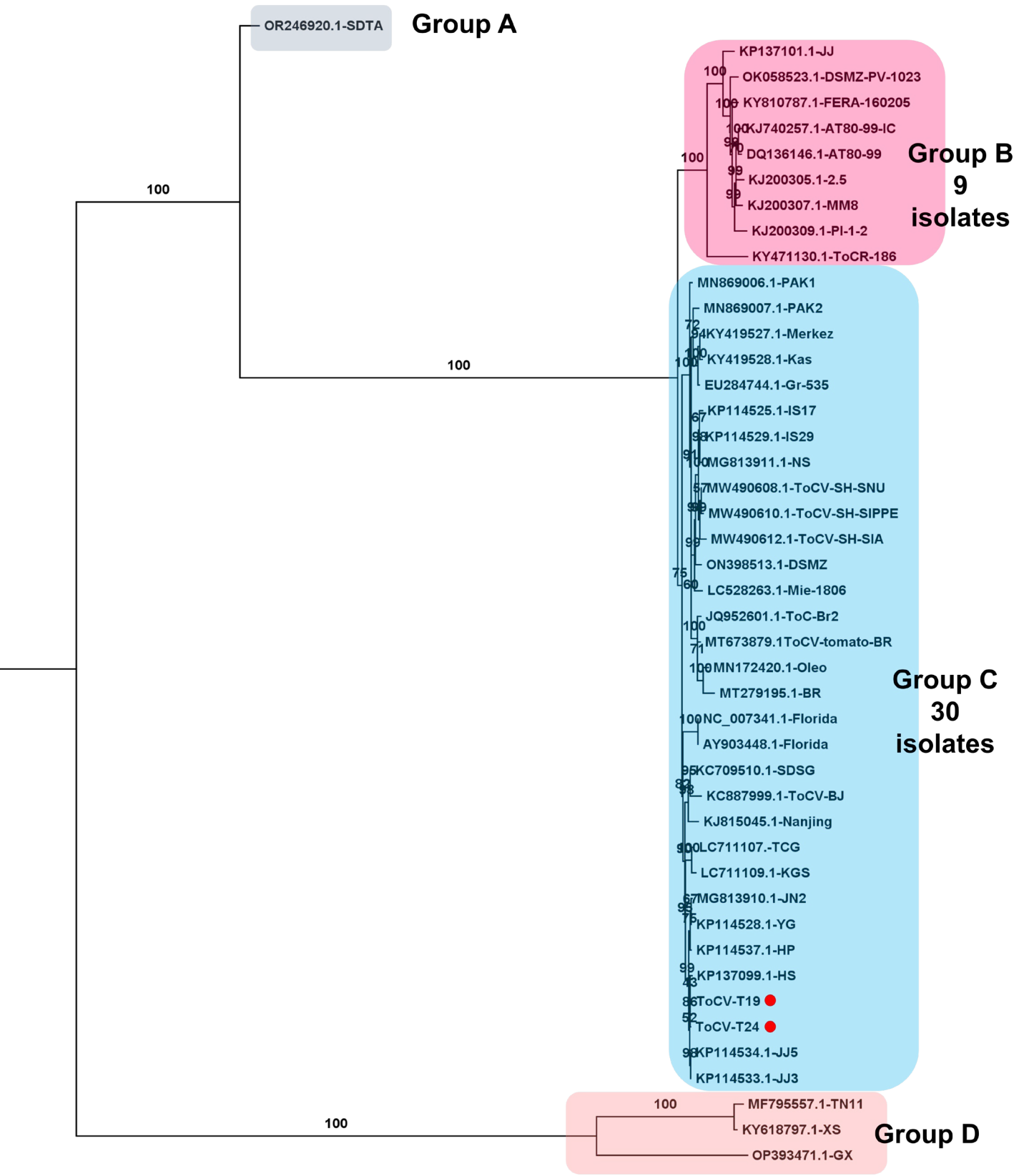

0.04

Supplement: Supplementary file 1 [file viruses-15-02139-s001.zip › Figure S1.pdf]
